# Supplementary material for: Association of Androgenic Regulation and MicroRNAs in Acinar Adenocarcinoma of Prostate
Source: Genes (Basel). 2022 Mar 30;13(4):622. doi: 10.3390/genes13040622 (PMC9030213; doi:10.3390/genes13040622)
Supplement: Supplementary file 1 [file genes-13-00622-s001.zip › genes-1618224-supplementary.pdf]

Supplementary Table S1. Clinical data of patients with CaP stratified for the D'Amico score.

D'AMICO Score	Low (n=11)	Intermediate (n=24)	High (n=5)
Age[a]	72.2	66	59.6
PSA Level*[a]	5.7	10	131.4
Gleason score	≤ 6(20%)	7 (20%)	≥8(60%)
Clinical staging	T2b(40%)<br>T2c(60%)	T2a(18.2%)<br>T2b(4.5%)<br>T2c(77.3%)	T2c(100%)

*Preoperative PSA; [a]Median.

Supplementary Table S2. Comparative prostatic expression of miRs (27a-3p; 124; 130a; 488-3p; 506) and AR molecular markers between the CaP and NPH groups.

Molecular markers	NPH (n=41)*	CaP (n=43)*	P value
**miR27a-3p** [a]	0.99 (0.96-1.02)	2.01 (1.86-2.19)	**3.06×10^{-15}**
**miR124** [a]	1.07 (1.07-1.07)	0.67 (0.65-0.68)	**2.78×10^{-15}**
**miR130a** [a]	1.01 (1.00-1.01)	0.50 (0.46-0.54)	**3.22×10^{-15}**
**miR488-3p** [a]	1.00 (1.00-1.01)	0.69 (0.66-0.72)	**3.04×10^{-15}**
**miR506** [a]	0.93 (0.91-0.94)	0.66 (0.65-0.66)	**3.04×10^{-15}**
**AR**[a]	0.94 (0.90-0.96)	1.71 (1.65-1.77)	**3.06×10^{-15}**

[a]Mann-Whitney Test; *μ(h); μ: median; h: variance.

Supplementary Table S3. Comparisons of Western Blot expression values of investigated miRs quantitative expression

miRNA	NPH[a]	CaP[a]	p-valor[b]
miR27a-3p	0.99 (0.96-1.02)	2.00 (1.84-2.17)	$2.74x10^{-15}$
miR124	1.07 (1.073-1.076)	0.66 (0.65-0.68)	$1.06x10^{-15}$
miR130a	1.01 (1.00-1.02)	0.49 (0.45-0.54)	$1.07x10^{-15}$
miR488-3p	1.00 (1.002-1.006)	0.69 (0.66-0.72)	$1.14x10^{-15}$
miR506	0.92 (0.90-0.94)	0.66 (0.65-0.67)	$2.99x10^{-15}$

[a]Median expression and IC95%; [b] Mann-Whitney Test

Supplementary Table S4. Sensitivity and specificity results for each coordinate point of the ROC curve for miRNA that tested positive if less than or equal to the cutoff point (miR124, miR130a, miR488-3p, miR506).

miRNA	Positive if less than or equal to[a]	Sensitivity	1 - Specificity
miR124	-0.394	0	0
	0.615	0.07	0
	0.639	0.186	0
	0.656	0.395	0
	0.662	0.581	0
	0.668	0.605	0
	0.675	0.674	0
	0.689	0.767	0
	0.733	0.907	0
	0.918*	1.000	0
	1.075	1.000	0.537
	2.080	1.000	1.000
miR130a	-0.68	0	0
	0.32	0.023	0
	0.322	0.047	0
	0.326	0.07	0
	0.334	0.093	0
	0.342	0.116	0
	0.345	0.14	0
	0.364	0.163	0
	0.389	0.209	0
	0.401	0.233	0
	0.405	0.256	0
	0.414	0.279	0
	0.422	0.349	0
	0.431	0.372	0
	0.439	0.395	0
	0.445	0.419	0
	0.4537	0.488	0
	0.46	0.512	0
	0.48	0.535	0
	0.496	0.558	0
	0.499	0.581	0
	0.505	0.605	0
	0.516	0.628	0
	0.547	0.651	0
	0.573	0.674	0
	0.578	0.698	0
	0.586	0.721	0
	0.594	0.744	0
	0.599	0.767	0
	0.617	0.791	0
	0.636	0.814	0
	0.651	0.837	0
	0.662	0.86	0
	0.667	0.884	0

	0.673	0.907	0
	0.688	0.93	0
	0.714	0.953	0
	0.798*	0.977	0
	0.903	0.977	0.024
	0.973	1.000	0.024
	1.015	1.000	0.659
	2.020	1.000	1.000
**miR488-3p**	-0.459	0	0
	0.546	0.023	0
	0.557	0.093	0
	0.565	0.116	0
	0.566	0.14	0
	0.586	0.163	0
	0.608	0.186	0
	0.615	0.209	0
	0.621	0.233	0
	0.624	0.279	0
	0.638	0.302	0
	0.658	0.326	0
	0.667	0.349	0
	0.673	0.372	0
	0.682	0.395	0
	0.691	0.419	0
	0.697	0.465	0
	0.705	0.488	0
	0.712	0.535	0
	0.718	0.581	0
	0.725	0.605	0
	0.734	0.628	0
	0.741	0.651	0
	0.752	0.674	0
	0.763	0.698	0
	0.768	0.767	0
	0.772	0.791	0
	0.775	0.837	0
	0.783	0.86	0
	0.790	0.907	0
	0.799	0.93	0
	0.826	0.953	0
	0.847	0.977	0
	0.925*	1.000	0
	1.005	1.000	0.585
	1.020	1.000	0.976
	2.030	1.000	1.000
	-0.388	0.000	0.000
	0.618	0.023	0.000
	0.625	0.047	0.000

	0.627	0.070	0.000
	0.630	0.093	0.000
	0.632	0.116	0.000
	0.636	0.140	0.000
	0.639	0.163	0.000
	0.639	0.186	0.000
	0.645	0.256	0.000
	0.651	0.279	0.000
	0.652	0.326	0.000
	0.652	0.349	0.000
	0.653	0.372	0.000
	0.656	0.395	0.000
	0.659	0.465	0.000
	0.662	0.488	0.000
	0.664	0.558	0.000
**miR506**	0.666	0.581	0.000
	0.671	0.605	0.000
	0.675	0.651	0.000
	0.677	0.698	0.000
	0.678	0.744	0.000
	0.682	0.791	0.000
	0.689	0.837	0.000
	0.691	0.884	0.000
	0.694	0.953	0.000
	0.697	0.977	0.000
	0.759*	1.000	0.000
	0.825	1.000	0.049
	0.845	1.000	0.122
	0.865	1.000	0.146
	0.875	1.000	0.220
	0.890	1.000	0.268
	0.905	1.000	0.293
	0.915	1.000	0.366
	0.925	1.000	0.439
	0.935	1.000	0.537
	0.945	1.000	0.585
	0.955	1.000	0.610
	0.965	1.000	0.707
	0.975	1.000	0.756
	0.985	1.000	0.829
	0.995	1.000	0.902
	1.015	1.000	0.951
	1.035	1.000	0.976
	2.040	1.000	1.000

a. The smallest cut-off value is the minimum observed test value minus 1 and the largest cut-off value is the maximum observed test value plus 1. All other cutoff values are the means of two consecutive ordered observed test values. *Defined cutoff point with better sensitivity and specificity

Supplementary Table S5. Sensitivity and specificity results for each coordinate point of the ROC curve for miRNA that tested positive if greater than or equal to the cutoff point (miR27a-3p).

MiRNA	Positive if greater than or equal to[a]	Sensitivity	1 - Specificity
	-0,11	1,000	1,000
	0,895	1,000	0,976
	0,905	1,000	0,902
	0,915	1,000	0,683
	0,93	1,000	0,585
	0,96	1,000	0,488
	1,005	1,000	0,463
	1,055	1,000	0,439
	1,085	1,000	0,22
	1,095	1,000	0,024
	1,217	1,000	0
	1,341	0,977	0
	1,368	0,953	0
	1,413	0,93	0
	1,438	0,907	0
	1,439	0,884	0
	1,473	0,86	0
	1,508	0,837	0
	1,544	0,814	0
	1,629	0,791	0
	1,684	0,767	0
	1,689	0,744	0
	1,696	0,721	0
**miR27a-3p**	1,703	0,698	0
	1,746	0,674	0
	1,813	0,651	0
	1,843	0,628	0
	1,872	0,605	0
	1,912	0,581	0
	1,933	0,558	0
	1,942	0,535	0
	1,948	0,488	0
	1,954	0,419	0
	1,972	0,395	0
	1,999	0,372	0
	2,022	0,349	0
	2,040	0,326	0
	2,116	0,302	0
	2,257	0,279	0
	2,340	0,256	0
	2,364	0,233	0
	2,381	0,209	0
	2,387	0,186	0
	2,434	0,163	0
	2,483	0,116	0
	2,491	0,093	0
	2,518	0,07	0

	2,795	0,047	0
	3,697	0,023	0
	5,345	0	0

[a]The smallest cutoff value is the minimum observed test value minus 1, and the largest cutoff value is the maximum observed test value plus 1. All other cutoff values are the means of two consecutive ordered observed test values.

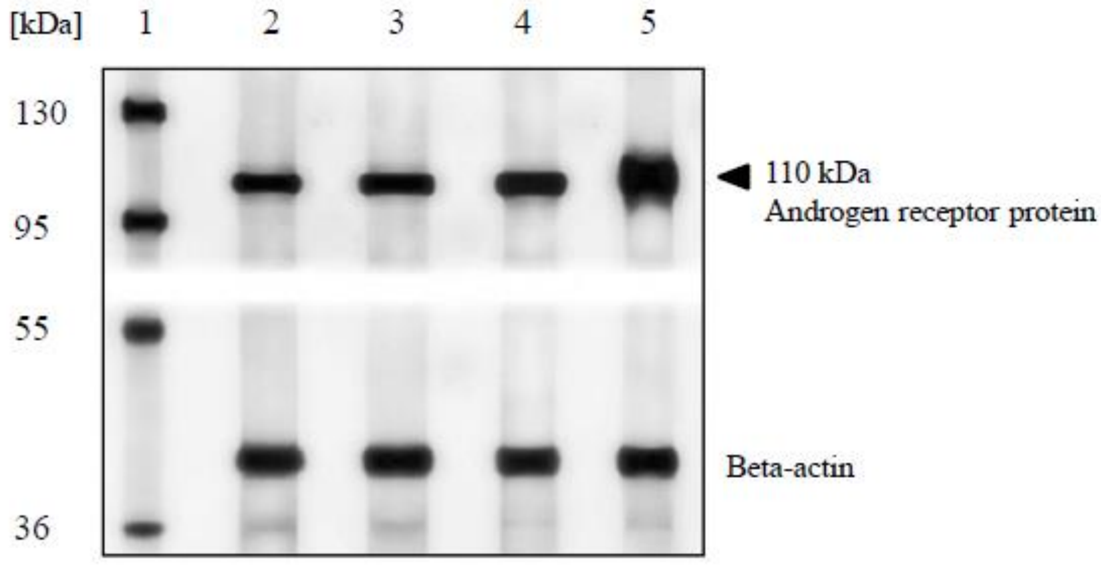

Supplementary Figure S1. Western Blot. Lane 1: Marker [kDa] 130, 95, 55, 36. Lane 2: Corresponds to a pool of Androgen Receptor proteins (AR protein) extracted from 41 nodular prostatic hyperplasia (NPH) tissues were designated as a calibrator from each tumor. Lane 3: Correspond to patient with Prostate Adenocarcinoma (CaP) with Gleason 4+5, pT2bN0; and PSA 12.7. Lane 4: Correspond to patient with CaP with Gleason 4+3, pT2cN0 and PSA1 16.9. Lane 5: Correspond to patient with CaP with Gleason 5+4, pT3bNO and PSA1 28.7.
